# Inoculation of Scrapie with the Self-Assembling RADA-Peptide Disrupts Prion Accumulation and Extends Hamster Survival

**DOI:** 10.1371/journal.pone.0004440

**Published:** 2009-02-12

**Authors:** Robert Hnasko, Cathrin E. Bruederle

**Affiliations:** United States Department of Agriculture (USDA), Agricultural Research Service (ARS), Pacific West Area (PWA), Western Regional Research Center- Foodborne Contaminants Research Unit (WRRC-FCR), Albany, California, United States of America; Massachusetts General Hospital and Harvard Medical School, United States of America

## Abstract

Intracerebral inoculation of 263K Scrapie brain homogenate (PrPsc) with a self-assembling RADA-peptide (RADA) significantly delayed disease onset and increased hamster survival. Time of survival was dependent on the dose of RADA and pre-incubation with PrPsc prior to inoculation. RADA treatment resulted in the absence of detectable PrPsc at 40 d followed by an increased rate of PrPsc accumulation at 75 d up to sacrifice. In all PrPsc inoculated animals, clinical symptoms were observed ∼10 d prior to sacrifice and brains showed spongiform degeneration with Congo red positive plaques. A time-dependent increase in reactive gliosis was observed in both groups with more GFAP detected in RADA treated animals at all time points. The PrP protein showed dose-dependent binding to RADA and this binding was competitively inhibited by Congo Red. We conclude that RADA disrupts the efficacy of prion transmission by altering the rate of PrPsc accumulation. This is the first demonstration that a self-assembling biomolecular peptide can interact with PrPsc, disrupt the course of Scrapie disease process, and extend survival.

## Introduction

Transmissible spongiform encephalopathies are incurable, fatal neurodegenerative diseases characterized by the accumulation of abnormal prion protein (PrPsc), neuronal cell death and vacuolation of brain [1]. The PrPsc protein is extractable from diseased tissue and is distinguished from endogenous PrPc by partial protease resistance and detergent insolubility [2]. The transmissible agent is the PrPsc protein and it serves as a template for the molecular conversion of endogenous PrPc into the abnormal PrPsc structural isoform [1]. Host expression of PrPc is necessary for disease transmission, as ablation of the PrPc gene prevents disease [3] and over expression of PrPc followed by PrPsc challenge accelerates disease [4]. The molecular events that mediate neuronal PrPc to PrPsc conversion, not simply accumulated PrPsc, appears to be the initiating factor mitigating the neurodegenerative disease process [5]. Yet the mechanism responsible for the conformational conversion of PrPc to an infectious prion remains enigmatic.

The use of synthetic peptides designed with intrinsic functional domains have been engineered to facilitate drug delivery, cell attachment, and tissue regeneration [6]–[8]. These biomolecular scaffolds exploit the properties of natural protein sequences to mediate targeted cellular events [9]. They offer distinct advantages over traditional pharmacotherapies because they are composed of normal biological constituents, devoid of animal contaminants, and are biodegradable [10]. Moreover, some of these substrates can mimic the dynamic structural changes observed in protein mis-folding diseases [11], [12]. The use of these peptides to inhibit endogenous protein mis-folding may prove useful in the elucidation of molecular conversion events or therapeutic intervention.

In this report we identify a self-assembling synthetic peptide composed of a 16-mer RADA repeat that significantly extends hamster survival when pre-incubated with 263K Scrapie prior to intracerebral inoculation. RADA combined with Scrapie results in an initial delay in detectable PrPsc followed by increased accumulation at later time points. There is a concomitant delay in observable Scrapie symptoms despite elevated PrPsc levels at 75-days when equivalent control animals required sacrifice. Moreover, RADA treatment shows a similar time-course for the induction of reactive gliosis and GFAP as PrPsc alone, but with increased levels at all time points. Furthermore, we show dose-dependent binding of PrP to RADA and demonstrate competitive binding inhibition of PrP to RADA with the amyloid-binding dye Congo red. We postulate that a physiochemical interaction of PrPsc with RADA impedes the efficacy of prion conversion and disease progression by disrupting the rate of PrPsc accumulation through altered clearance and distribution.

## Results

### Increased survival time in animals inoculated with PrPsc and RADA-peptide

**Figure 1:**
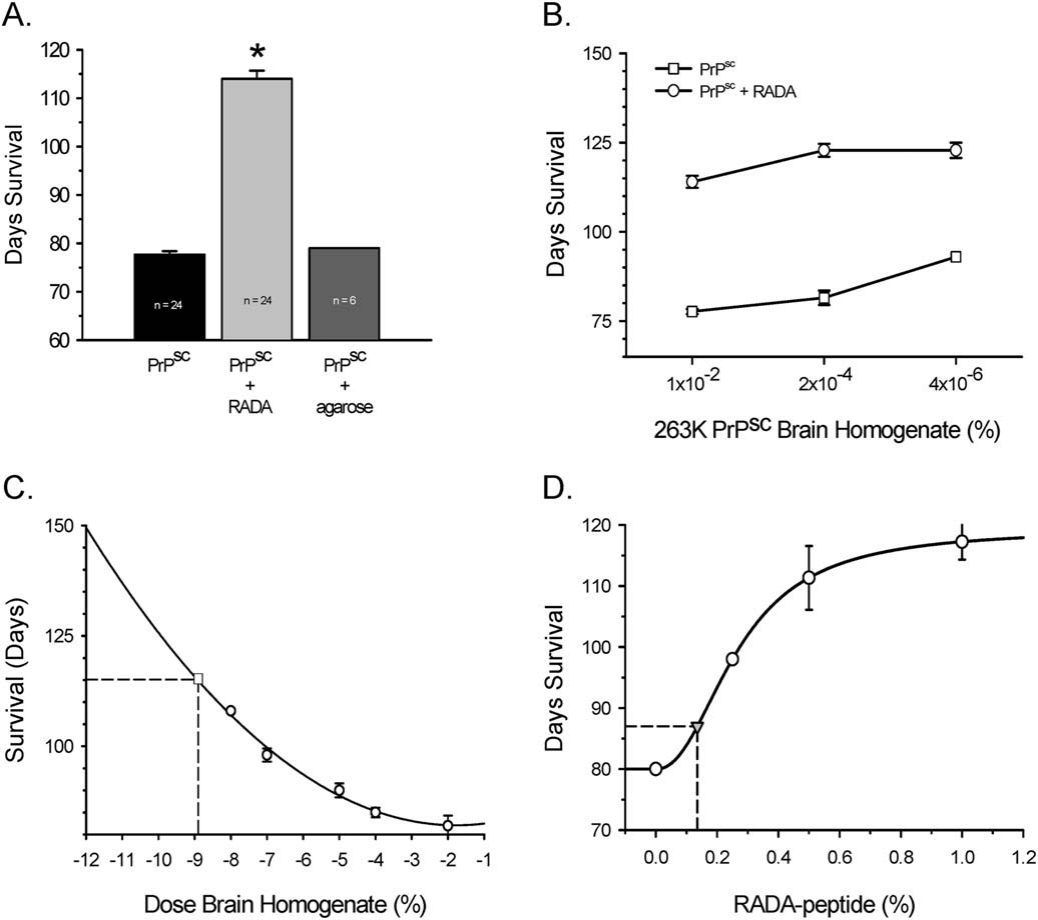
RADA promotes increased survival of hamsters inoculated with 263K Scrapie. *Fig. 1A* compares hamster survival in days (mean±SEM) following intracerebral inoculation with 1% Scrapie alone (PrPsc), Scrapie pre-incubated with 0.9% w/v RADA (PrPsc+RADA) or Scrapie agarose plugs (PrPsc+agarose). *Fig. 1B* compares hamster survival in days (mean±SEM) with increased dilution of Scrapie brain homogenate alone (open squares) to equivalent doses of Scrapie pre-incubated with 0.9% RADA (open circles). *Fig. 1C* depicts a hamster survival curve in days (N = 6; mean±SEM) with increasing dilution of Scrapie inoculant (open circles). Plotted on the curve (open square) is the mean survival in days of hamsters inoculated with 10^−2^ Scrapie combined with 0.9% RADA depicting equivalent titer of Scrapie inoculum. *Fig. 1D* shows a dose-dependent increase in hamster survival in days (mean±SEM) with increasing concentration of RADA (open circles) inoculated with 1% Scrapie. A synthetic 16-mer RADA-peptide was hydrated combined with 1% Scrapie and inoculated. Survival in days was plotted (grey triangle) and used to estimate RADA concentration.

Hamsters inoculated with PrPsc combined with RADA survived significantly longer than those that received an equivalent dose of PrPsc alone. The mean survival time of animals inoculated with 1% PrPsc (10^−2^ dose) combined with 0.9% RADA was 114 d (n = 24) as compared to 78 d (n = 24) for animals that received equivalent PrPsc alone or 80 d (n = 6) for animals inoculated with PrPsc-agarose plugs (Fig. 1A; P<0.001). A rapid toxicity was observed with this combined inoculum in ∼20% of the hamsters within 24 h (Table 1). This toxicity did not occur in animals that received equivalent doses of PrPsc alone, PrPsc combined with agarose, RADA alone, or normal brain homogenate combined with RADA. With increased dilution of PrPsc+RADA there was reduced morbidity in that 24 h period. No animals died unexpectedly after this initial 24 h period.

**Table 1:** Effect of RADA on Mortality within 24 h.

Inoculum (ic)	0.9% RADA	N	Loss <24 h (N)
RADA alone	**+**	12	0
1% PrPc	**−**	16	0
1% PrPc	**+**	26	0
1% PrPsc	**−**	74	0
1% PrPsc	Agarose	6	0
1% PrPsc	**+**	43	9
0.02% PrPsc	**+**	8	2
0.0004% PrPsc	**+**	8	1

Mortality data at <24 h following single unilateral intracerebral (ic) inoculation of brain homogenates and RADA. Normal (PrPc) or Scrapie (PrPsc) infected hamster brain homogenates were inoculated alone (−) or with 0.9% w/v RADA (+). Low melt Agarose (0.9% w/v) combined with PrPsc served as an additional control. RADA alone or combined with PrPc brain homogenate resulted in no lethality. Combined RADA with PrPsc resulted in a rapid mortality within 24 h in ∼20% of the animals inoculated. N = number of hamsters inoculated.

As expected, increased dilution of PrPsc resulted in a dose-dependent increase in survival time (Fig. 1B; squares). Importantly, increased dilution of PrPsc with 0.9% RADA resulted in a significant increase in survival time as compared to the equivalent doses of PrPsc alone (Fig. 1B; circles; Mann-Whitney P<0.01). The combined inoculum showed dose-dependence, but a plateau in total survival time (∼125 d) was observed at dilutions of PrPsc below 2×10^−4^, suggesting that maximal survival promoted by RADA had been achieved. Based on our survival curve for titrated 263K Scrapie (Fig. 1C; open circles; n = 6 each data point) the initial PrPsc inoculum of 10^−2^ when combined with RADA was equivalent to a starting inoculum of 10^−9^ PrPsc alone, a 7-log reduction in prion titer (Fig. 1C; dashed line). In all groups inoculated with PrPsc, clinical Scrapie symptoms (startle response followed by increased righting reflex and ataxia) began 10–15 d prior to sacrifice (data not shown). No animals injected with RADA alone or combined with normal brain homogenate exhibited Scrapie symptoms and those not sacrificed as age-matched controls survived >200 days.

### Increased survival is RADA-peptide dose-dependent

Increased survival time of hamsters inoculated with PrPsc was RADA dose-dependent. When RADA concentration was varied 0–1% with a constant 10^−2^ dose of PrPsc there was a dose-dependent increase in survival time with maximal survival achieved at the highest concentration of RADA used (Fig. 1D; circles). The survival curve was sigmoid suggesting that no additional increase in survival would be achieved with RADA concentrations above 1% when combined with the 10^−2^ PrPsc dose. We synthesized our own 16-mer RADA-peptide to 95% purity and estimated the concentration of our preparation to be ∼0.2% w/v. This material when combined with PrPsc increased survival to ∼87 d from 80 d compared to PrPsc alone (Mann-Whitney; P = 0.02) and RADA concentration calculated from our survival curve was ∼0.14% (Fig. 1D; triangle).

### Total PrP, Prion and GFAP proteins are elevated in brain of animals inoculated with PrPsc -RADA

**Figure 2:**
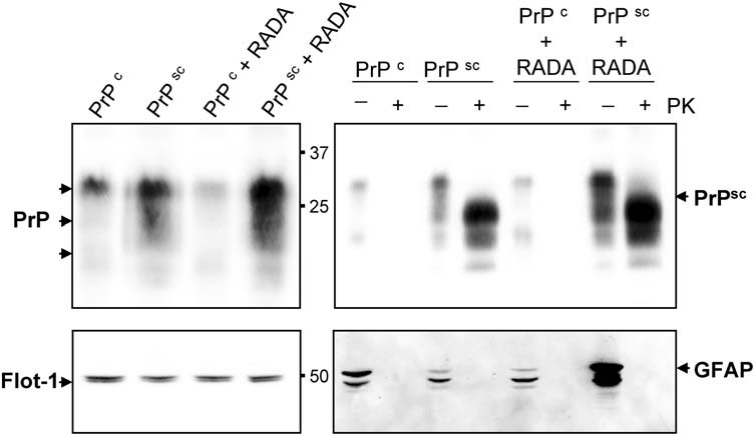
Prion and GFAP proteins are increased in brain homogenates from hamsters inoculated with Scrapie+RADA. Total PrP protein was detected by Western blot in hamster brain homogenates (20 µg/lane) from normal (PrPc; 75 d), Scrapie infected (PrPsc; 75 d), normal combined with RADA (PrPc+RADA; 115 d), and Scrapie combined with RADA (PrPsc+RADA; 115 d). A prominent PrP band was detected at ∼30 kDa and two lower molecular weight bands at ∼22 kDa and 19 kDa (top left panel). Flotillin-1 (Flot-1) was used as a loading control and a doublet was detected at ∼45 kDa (bottom left panel). Digestion of brain homogenates with proteinase-K (+PK) demonstrated the presence of prion protein in the PrPsc and PrPsc+RADA samples, but not in PrP^c^ or PrPc+RADA. A detectable MW shift was observed for PrPsc in all three bands with a shift from 30 kDa to 25 kDa occurring for the predominant band (top right panel). A notable increase in PK-resistant prion was detected in the PrPsc+RADA compared to the PrPsc alone. Detection of GFAP was used to verify the complete PK-digestion of samples (bottom right panel). Three detectable GFAP bands were resolved (∼50, 45 and 40 kDa) in the non-PK treated samples with increased detection of GFAP in PrPsc+RADA brain.

Western blot showed increased detection of total PrP protein in brain homogenate of animals that received combined PrPsc with RADA (Fig. 2, top left panel) at the time of sacrifice. Both PrPsc alone and PrPsc combined with RADA had increased PrP protein compared to animals treated with normal brain homogenate (PrPc). This increase in total PrP is a measure of both PrPc and accumulated PrPsc. The highest level of PrP was detected in brain homogenates from animals treated with PrPsc combined with RADA. Protein load was normalized by BCA and confirmed by detection of flotillin-1 which remained unchanged (Fig. 2; bottom left panel). As expected, PrP from hamsters inoculated with control brain homogenate, either alone (PrPc) or in combination with RADA (PrPc+RADA), were proteinase-K (PK) sensitive (Fig. 2, top right panel). Brain homogenates from hamsters inoculated with PrPsc alone (PrPsc) or combined with RADA (PrPsc+RADA) had detectable PK-resistant prion, with the greatest amount detected in brain homogenate from animals that received the combined inoculant (Fig. 2, top right panel). Glial fibrillary acidic protein (GFAP) was used to validate the fidelity of the PK reaction and is completely digested by PK in all groups (Fig. 2, bottom right panel). Interestingly, more detectable GFAP protein was present in brain homogenate from the animals that received PrPsc+RADA (Fig. 2, bottom right panel).

### Delayed PrPsc accumulation with RADA-peptide

**Figure 3:**
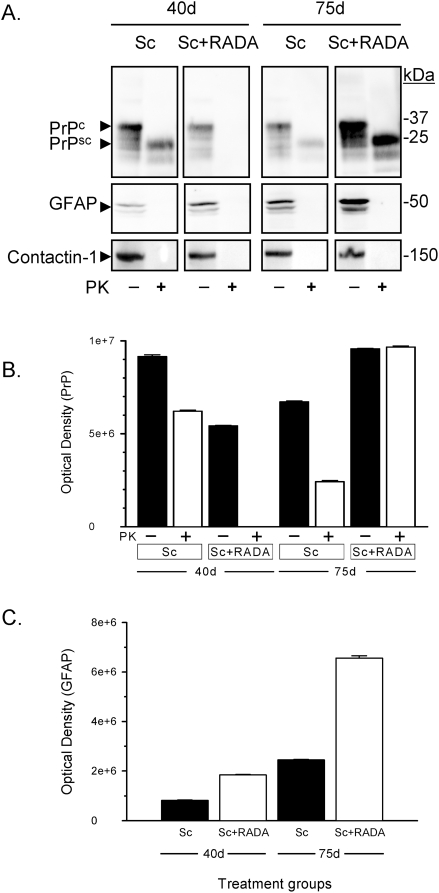
Time-dependent increase in GFAP and PrP protein in hamsters inoculated with combined Scrapie+RADA. *Fig. 3A* shows a Western blot from hamster brain homogenates inoculated with 1% Scrapie alone (Sc) or combined with 0.9% RADA (Sc+RADA) at 40 d and 75 d treated with (+) or without (−) proteinase-K (PK). The top panels show PrPc and PrPsc detection at each time point (20 µg). Middle panels show detection of GFAP protein and bottom panel contactin-1. *Fig. 3B* shows quantification of total PrP (−PK) and prion (+PK) from Western blots at each time-point. *Fig. 3C* show quantification of ∼55 kDa GFAP band (−PK) from Western blots from each time-point.

In contrast to animals that received inoculation of PrPsc alone, no PK-resistant prion was detected by Western blot in brain homogenates at 40 d from animals that received PrPsc+RADA (Fig. 3A; top panels; left side). At 75 d the animals that received PrPsc alone had late stage clinical Scrapie symptoms whereas those that received PrPsc with RADA had no observable symptoms (data not shown). At the 75 d time point PK-resistant prion was detected in brain of animals that received PrPsc alone or combined with RADA (Fig. 3A; top panel; right side). Detection of GFAP protein by Western blot increased from day 40 to 75 in animals of both treatment groups (Fig. 3A; middle panels), but was greater in brains of animals that received PrPsc+RADA at both time points (Fig. 3C; P<0.001). Protein load was normalized by BCA and confirmed by the equal and unchanged detection of the contactin-1 protein which also served to validate PK digestion (Fig. 3A; bottom panels). Quantification of Western blots shows at 40 d there is significantly more PK-resistant prion protein in animals inoculated with PrPsc alone (Fig. 3B; P<0.001), whereas at 75 d there is significantly more prion protein in brain homogenate from animals inoculated with PrPsc+RADA (Fig. 3B; P<0.001).

### RADA induced ventricular enlargement and delayed spongiform degeneration

**Figure 4:**
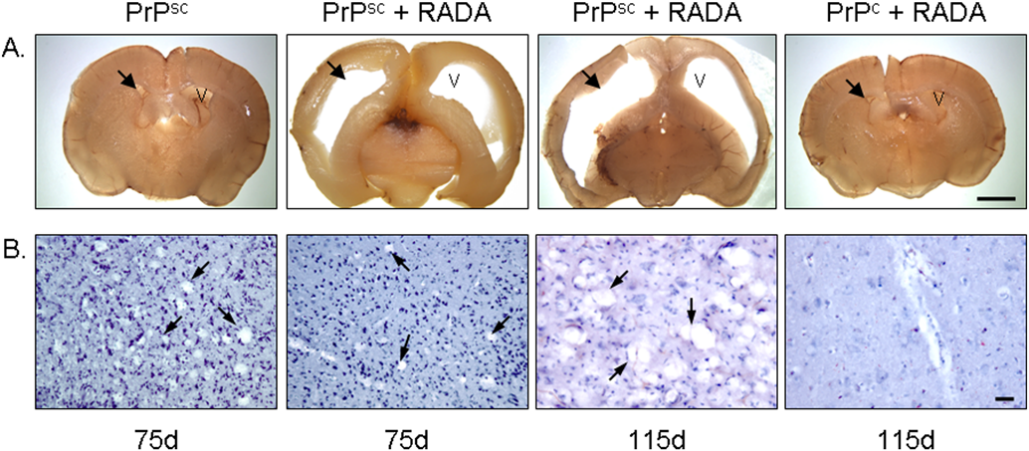
Combined inoculation of Scrapie+RADA results in ventricular enlargement and delayed spongiform degeneration. Brain sections were obtained from hamsters inoculated with 1% Scrapie brain homogenate alone (PrPsc) after 75 d, Scrapie combined with 0.9% RADA (PrPsc+RADA) after 75 d and 115 d and normal brain combined with RADA (PrPc+RADA) after 115 d. *Fig. 4A* shows gross morphology from coronal sections of whole hamster brains. No ventricular enlargement (black arrows) was observed in animals that received inoculation of PrPsc alone (top left panel) or PrPc+RADA (top right panel), whereas brains from hamsters inoculated with PrPsc+RADA had massive enlargement of lateral ventricles at both 75 d and 115 d (top middle panels). V = ventricle. Bar = 2 mm. *Fig. 4B* are micrographs from brain sections stained with Hematoxylin showing spongiform degeneration present in hamsters cortex following inoculation with PrPsc alone after 75 d (bottom left panel; arrows) and PrPsc+RADA after 75 d and 115 d (middle panels; arrows). No spongiform degeneration was detected in animals inoculated with PrPc+RADA after 115 d (bottom right panel). Bar = 50 µm.

At the time of sacrifice the brains of hamsters inoculated with PrPsc+RADA were filled with an increased amount of cerebral spinal fluid (CSF) compared to animals inoculated with PrPsc alone, however gross wet weight (∼1 g) did not differ significantly between these treatment groups (data not shown). Coronal brain sections showed enlarged lateral ventricles from animals that received PrPsc+RADA at 75 d and 115 d relative to those inoculated with PrPsc alone or PrPc+RADA (Fig. 4A; top panels). Typical spongiform degeneration was observed in cortical brain sections at 75 d from animals inoculated with Scrapie alone and to a lesser extent those inoculated with RADA (Fig. 4B; bottom panels). However, at 115 d the brain of PrPsc+RADA inoculated hamsters had abundant spongiform degeneration equivalent to levels observed at 75 d in animals inoculated with Scrapie alone. Importantly, no abnormal ventricular enlargement or spongiform degeneration was observed in brain sections from animals inoculated with PrPc+RADA. Hence, ventricular enlargement is a consequence of the combined action of PrPsc+RADA and not RADA alone. Moreover, our histopathology indicates that RADA delayed the progression of cortical spongiform degeneration induced by PrPsc.

### Increased reactive gliosis and PrP protein aggregates in brain following PrPsc inoculation with RADA-peptide

**Figure 5:**
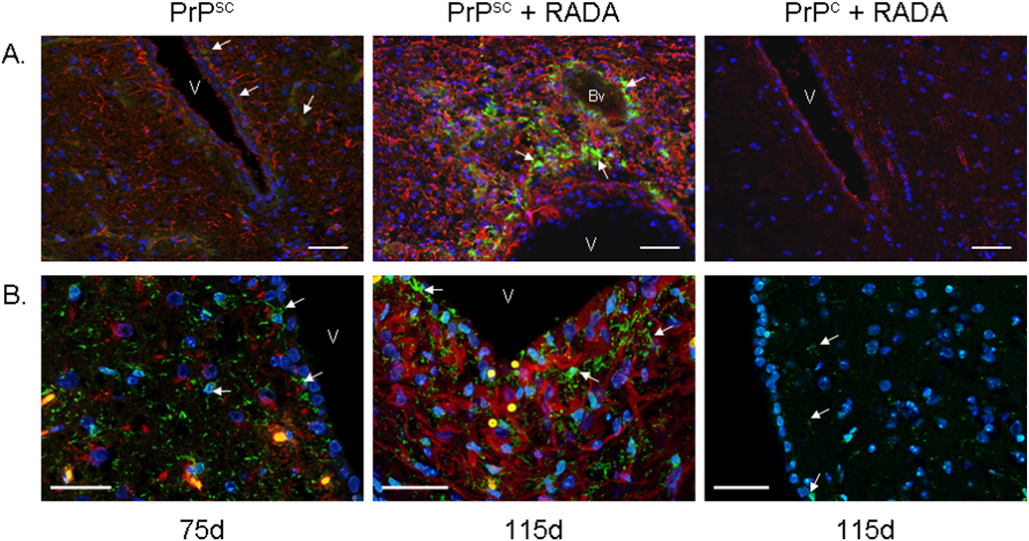
Scrapie induced reactive gliosis and PrP aggregation is increased by RADA. *Fig. 5A* shows dual labeled immunofluorescent GFAP (red) and PrP (green) hamster brain micrographs. Limited staining of GFAP and no detectable PrP was observed in PrPc+RADA treated brain after 115 d (top right panel). PrPsc treatment alone results in an increased detection of GFAP-positive astrocytes (red) and weak PrP protein (green, white arrows) after 75 d (middle panel). Combined PrPsc+RADA treatment (right panel) results in abundant and strong detection of both GFAP-positive astrocytes and large PrP deposits (green, arrows). V = ventricle. Bv = blood vessel. DAPI nuclei (blue). Bars = 50 µm. *Fig. 5B* shows micrographs of hamster brain sections labeled with GFAP (red) and IBA1 (green). Increased IBA1-positive immunoreactive microglia are detected in brain following inoculation of PrPsc alone after 75 d (left panel; white arrows) and PrPsc+RADA after 115 d (middle panel; white arrows) as compared to PrPc+RADA after 115 d (right panel; white arrows). IBA1 and GFAP immunoreactivity do not co-localize in dual labeled sections from PrPsc and PrPsc+RADA brain. V = ventricle. DAPI nuclei (blue). Bars = 50 µm.

Hamster brain sections from animals at the time of sacrifice showed increased immunoreactive GFAP-positive astrocytes following inoculation with PrPsc alone (day 75) or PrPsc+RADA (day 115) as compared to animals inoculated with PrPc+RADA (Fig. 5A; top panels). Few GFAP immunoreactive astrocytes were detectable at 115 d in control brain (PrPc+RADA) and PrP was undetectable by immunofluorescence. Animals that received PrPsc alone showed increased GFAP positive astrocytes with some detectable PrP protein; whereas those that received PrPsc+RADA showed a massive increase in immunoreactive GFAP and large PrP deposits. These immunoreactive PrP-positive aggregates were PK resistant (data not shown). In addition, PrPsc increased detection of the microglia/macrophage-specific calcium-binding protein IBA1 (ionized calcium binding adaptor molecule 1). Abundant immunoreactive IBA1-positive microglia were detected in 75 d PrPsc and 115 d PrPsc+RADA brain sections as compared to minimal detection in sections from animals inoculated with PrPc+RADA (Fig. 5B; bottom panels). The absence of IBA-1 and GFAP co-localization demonstrates that the reactive gliosis induced by PrPsc and PrPsc+RADA involves two distinct glial cell populations.

### PrP binds RADA-peptide and binding is inhibited by Congo Red

**Figure 6:**
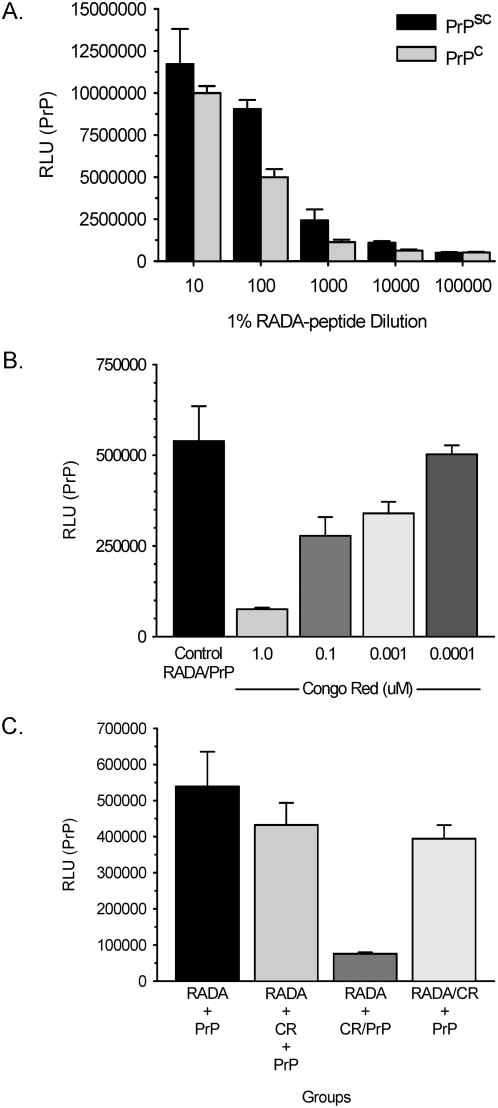
PrP binds RADA-peptide and binding can be inhibited with Congo red. *Fig. 6A* shows chemiluminescent detection of PrP binding to RADA dilutions from Scrapie (PrPsc) and normal (PrPc) brain homogenates. *Fig. 6B* shows quantification of PrP binding to RADA (Control RADA/PrP) following pre-incubation with dilutions of Congo Red. *Fig. 6C* compares the binding of PrP to RADA based on the order of reagent addition. Quantification of PrP binding to RADA: RADA followed by PrP (RADA+PrP), RADA followed by 1 µM Congo red (CR) then PrP (RADA+CR+PrP), RADA followed by pre-incubated PrP with 1 µM CR (RADA+CR/PrP), RADA pre-incubated with 1 µM CR followed by PrP (RADA/CR+PrP). RLU = relative light units.

Normal and Scrapie-infected brain homogenates were incubated with RADA bound to 96-well plates. PrP binding was dependent on the concentration of RADA with no significant difference observed between normal or Scrapie brain (Fig. 6A). Pre-incubation with Congo red resulted in a dose-dependent inhibition of binding of PrP to RADA with a significant inhibition of PrP binding observed at 1 µM Congo red (Fig. 6B; Mann-Whitney; P<0.001). Importantly, the inhibition of PrP binding to RADA by Congo red was dependent on the order of reagent addition; inhibition required Congo red pre-incubation with PrP prior to RADA exposure (Fig. 6C). RADA pre-incubated with Congo red before or after plate binding was ineffective in inhibiting the binding of PrP, suggesting that the Congo red interacts with PrP, not RADA, to inhibit binding.

### PrPsc infectivity is not modified by RADA-peptide

We used four independent preparations of RADA combined with PrPsc to show increased survival time in hamsters was not limited to a single sample. All treatment groups showed a significant increase in animal survival compared to those inoculated with equivalent dose of PrPsc alone (Table 2). Moreover, when brain homogenate was prepared from animals inoculated with PrPsc combined with RADA and re-inoculated back into a new group of hamsters (PrPsc/RADA; dose 10^−2^) no increase in survival was observed (Table 3). This demonstrates that the property of Scrapie infectivity was not modified by exposure to the RADA in subsequent passage. The same inoculant when combined with RADA again (PrPsc/RADA+RADA), resulted in an increased survival time.

**Table 2:** RADA Promotes Survival.

PrPsc+RADA	Mean	±SEM	N
Group 1	111	2.5	13
Group 2	120	2.4	5
Group 3	117	1.9	6
Group 4	102	4.3	10
Groups 1–4	110	2.0	34
PrPsc alone	78	0.7	24

Survival data following intracerebral inoculation of hamsters with 1% Scrapie brain homogenates (PrPsc) and 0.9% w/v RADA after 24 h. Four independent groups consisting of PrPsc brain homogenate combined with RADA (PrPsc+RADA) were evaluated for survival in days (Mean±SEM). The average survival time in days of all PrPsc+RADA (Groups 1–4) inoculated hamsters is compared to survival of those inoculated with equivalent doses of PrPsc alone. N = number of animals.

**Table 3:** Effect of RADA on Secondary Passage of Scrapie Brain Homogenate.

Treatment	MEAN	±SEM	N
PrPsc	78	0.7	24
PrPsc/RADA	80	0	6
PrPsc/RADA+RADA	117	1.9	6

Survival data following intracerebral inoculation of hamsters with 263K Scrapie brain homogenate following primary passage with RADA (PrPsc/RADA). Survival (Mean±SEM) of hamsters inoculated with 1% Scrapie alone (PrPsc) is compared to those inoculated with 1% PrPsc/RADA (homogenate derived from a 1% PrPsc+0.9% w/v RADA infected brain) and 1% PrPsc/RADA combined a second time with 0.9% w/v RADA. N = number of animals.

## Discussion

The complex molecular events that underlie most neurodegenerative diseases are poorly understood. In prion diseases the mechanism by which PrPc is converted to PrPsc is central to disease transmission and pathophysiology [1], [13]. Indeed, deposition of PrPsc in the absence of endogenous PrPc is insufficient to cause disease. Consequently, the disruption of PrPc to PrPsc conversion and concomitant cellular events provides a useful basis for therapeutic intervention.

The structural change from primarily alpha-helical PrPc to beta-sheet PrPsc is thought to underpin the aggregation status, protease resistance and replication capacity of PrPsc [14], [15]. The PrPsc template is necessary to promote conversion of PrPc in transmitted disease, but mutations in the PrP gene can impart intrinsic changes in PrPc primary structure that fosters spontaneous conformational conversion that leads to abnormal protein aggregation and prion diseases [1], [16]. The existence of multiple prion strains with differing biochemical and pathophysiological signatures suggests that a range of stabilized structural isoforms of PrPsc can be transmitted to PrPc [17], [18]. This functional diversity likely reflects the unique properties of stabilized prion structures and their ability to complex with distinct compliments of endogenous factors. Moreover, the neuropathological variability observed among prion strains may result from a selective targeting of a strain-specific PrPsc complex to unique cellular or extracellular sites within the brain [17], [19], [20]. Hence, disruption of the prion structure or its interaction with these endogenous factors would impede PrPc conversion, cellular distribution and neuropathology.

The disparity observed in the bioactivity of anti-prion agents such as polyene antibiotics and tricyclic compounds among prion strains may reflect their differing ability to disrupt PrPsc interaction with strain specific proteins [21]. These agents likely interrupt innate PrPsc+ protein interaction thereby modifying PrPsc distribution and associated cellular conversion events. These changes might reduce the rate or site of PrPsc accumulation thereby decreasing efficacy of prion transmission [20], [22]. However, impermanent changes to PrPsc structure would inexorably lead to its replication from PrPc and eventual disease. This may explain the ability of anti-prion agents to reduce the rate of PrPsc accumulation and extend animal survival, but not cure disease. Synthetic peptides derived from the primary sequence of PrPc have been shown to have anti-prion activity [23]. Peptides generated against PrPc residues 106–126 or 109–141 prevented PrPsc conversion in cell-free systems [24], [25]. Additionally, the use of PrP119–136 peptide was effective in decreasing PrPsc in chronically infected cells [26]. Soto *et al* generated a peptide corresponding to the amino acid residues of PrPc with the propensity to form beta-sheet structure and inserted incremental proline residues to disrupt the conformational requirements of an ordered beta-sheet. Pre-incubation of this peptide (iPrP13) with mouse-adapted Scrapie prior to intracerebral inoculation increased survival time in mice. Moreover, iPrP13 was able to reduce PrPsc in cultured cells following exposure to Scrapie infected brain homogenate [23]. This data demonstrates that a competitive peptide mimetic can impede PrPc to PrPsc conversion, result in reduced PrPsc accumulation and delay disease progression. Alternatively, transgenic mice expressing mutant PrP(P101L) at low level persist as clinically normal but can be induced to develop neurodegeneration and amyloid deposits by inoculation of a synthetic beta-sheet peptide (PrP89–143) carrying the P101L substitution [27]. These results suggest that a peptide mimetic can also induce PrPc conversion to PrPsc and promote disease.

Here we report the anti-prion activity of a synthetic RADA-peptide repeat that is unrelated to PrPc primary structure and self-assembles into a hydrated beta-sheet nanofiber matrix [11]. The RADA hydrogel provides a suitable substrate for neuron attachment, outgrowth, and regeneration [8], [28]. RADA likely mimics the adhesive proteins present in the extracellular matrix (ECM) that contain the RGD tri-peptide motif and modulate cell recognition, adhesion and migration [29]. Indeed, the core RAD motif in the RADA-peptide is similar to the ubiquitous integrin receptor binding site RGD [30], [31]. The PrP protein has been shown to interact with the ECM protein laminin, which contains a RGD domain and has self-assembling properties [32], [33]. Additionally, PrP interacts with the 37 kDa/67 kDa laminin receptor which plays a role in the propagation of Scrapie in vitro [34]. The increase in survival of rodents treated with sulfated glycans after Scrapie infection is likely mediated by disruption of prion/laminin receptor interaction [35]. Interestingly, an inverted RGD domain is found in the rodent PrP protein at the C-terminus (DGR aa 229–227) that is absent in other species.

Our data shows that pre-incubation of RADA with PrPsc brain homogenate prior to inoculation is necessary for its anti-prion activity. Moreover, we show that both PrPc and PrPsc bind RADA and binding can be competitively inhibited with Congo red. The planar dye Congo red has anti-prion activity and is routinely used as an amyloid stain [36]. We also show that Congo red binding competes with RADA for PrP binding and not by binding RADA directly. This suggests that Congo red and RADA share a common binding motif in PrPsc and implies a similar mechanism by which they exert their anti-prion activity. Although the mechanism by which Congo red binds amyloid and PrPsc has not been elucidated, its anti-prion activity may be mediated through stabilization of the PrPsc conformation that then impedes the process required for PrPc conversion [37], [38]. Interestingly, Congo red has a strong self-assembling activity, a property that other symmetric or planar dyes may share, in that they form ribbon-like micellar species that may provide an adhesive structure for binding beta-sheet peptide chains [39]. The symmetrical self-assembled beta-sheet rich RADA-scaffold may provide a complimentary surface for PrPsc binding and consequently uncoupling the process necessary for PrPc to PrPsc conversion.

Alternatively, the interaction of PrPsc with RADA may competitively disrupt PrPsc binding to endogenous ECM proteins. This could result in altered clearance of the PrPsc+RADA complex or a distinct extracellular distribution. A change in clearance of PrPsc mediated by RADA binding could result in a reduced PrPsc titer retained in the brain that would account for the delay in prion accumulation at 40 d and the increased survival. Yet, this does not explain the progressive increase in reactive gliosis from the time of inoculation, the increased rate of PrPsc accumulation from 40- to 75 d with a disconnect between the severity of clinical disease and total PrPsc. However, this could be explained by an altered extracellular distribution of the PrPsc-RADA complex. Localization to secondary sites not normally involved in PrPc to PrPsc conversion could act to sequester the PrPsc from it preferred neuronal site. Here the PrPsc+RADA complex could be more susceptible to protease degradation within the extracellular matrix or via intracellular pathways. Indeed, the conversion of PrPc to PrPsc can occur at multiple cellular sites [40], [41]. If a predominant, neuronal site is required for the neuropathological impact of prion disease then accumulation at secondary sites, such as glia, could alter the neuropathology. The progressive increase in reactive gliosis observed in animals inoculated with PrPsc+RADA suggests an active disease process but may reflect a more robust neuro-protective response.

Future experiments will determine binding of prion from different species and strains to RADA and the capability of RADA to disrupt *in vitro* PrPc to PrPsc conversion. The participation of an intrinsic rodent RGD-like motif in PrPc+ protein interaction might account for discrepancies in the efficacy of prion conversion between species, strains, and anti-prion compounds observed in assays based on the rodent PrP protein. A further evaluation of PrP interaction with synthetic peptides corresponding to RGD motifs may be useful in elucidating the molecular mechanism by which certain compounds exert their anti-prion activity and illuminate distinct biochemical properties inherent in the structural variability of prions. We conclude that RADA impedes the propagation of PrPsc from endogenous PrPc resulting in an altered rate of PrPsc accumulation that delays disease onset and extends survival.

## Materials and Methods

### Animals

Female Syrian Golden hamsters (LVG) at 4 weeks of age were housed in pairs on a 12 h light-dark cycle. Animals were provided continual access to food and water. All protocols were approved by the USDA animal care and use committee and experimental procedures conducted in certified BL2 laboratory. PrPsc-infected hamsters were sacrificed when clinical Scrapie symptoms included; increased startle response, ataxia, and >5 s righting reflex.

### Reagents

Infectious 263K Scrapie was propagated by serial passage in hamster brains for 72 days following intracerebral inoculation. Infected or normal brains were frozen on dry ice and stored at −80 C until use. A 16-mer RADA peptide hydrogel was purchased from BD Bioscience as Puramatrix (Product #354250; Lot#425714) or synthesized to 95% purity (AnaSpec, CA) for comparison. Congo red (Sigma-Aldrich, MO) was solubalized in water at 1 mM. Ultra pure low melting temperature agarose (AquaPor LM, National Diagnostics, GA) was dissolved in water at 65 C and combined with brain homogenate to form agarose plug controls.

### Inoculation

Hamster brain homogenates (10%) were prepared by homogenization in 320 mM sucrose and pre-cleared by centrifugation at 3000 RPM for 10 min at 4 C. Resulting supernatant was processed for inoculation by 1∶10 dilution in 320 mM sucrose or PuraMatrix (1% w/v synthetic 16-mer RADA peptide hydrogel) to yield a 1% brain homogenate (dose equivalent 10^−2^) in 0.9% peptide hydrogel. Additionally, brain homogenate or PuraMatrix was serially diluted in 320 mM sucrose and final concentration of brain homogenate was achieved by 1∶10 dilution in PuraMatrix. Samples were vortexed and incubated at room temperature for 20 min prior to unilateral intracerebral inoculation (40 µl) using a 27-guage needle attached to a 1 ml syringe.

### Western Blotting

Hamster brain homogenates were processed by homogenization in a MES buffer (25 mM MES, 150 mM NaCl, 60 mM n-octyl-glucoside and 1% Triton X-100, pH 6.5) at 4 C and protein concentration quantified by BCA (Pierce, IL). Proteinase-K treatment of 25 µg protein was performed at 60 C for 1 h at a final concentration of 15 µg/ml followed by heat denaturation in sample buffer. Equal concentration of protein (20 µg) were loaded on 4–12% polyacrylamide gels and subjected to electrophoresis, transferred to nitrocellulose and probed with anti-PrP mAb IPC1 at 1∶10K (Sigma-Aldrich), rbt-a-GFAP 1∶5K (Novus, CO), gt-a-flotillin-1 at 1∶1K (H-104; Santa Cruz, CA) or gt-a-contactin-1 at 1∶200 (S-20; Santa Cruz). Corresponding secondary antibodies were used; gt-a-m-HRP (Pierce, IL), gt-a-rbt-HRP (Pierce), or dnk-a-gt-HRP (Jackson, PA) at 1∶10K and blots resolved by ECL (Supersignal Pico, Pierce) and imaged on Flurochem HD documentation system (Alpha Innotech, CA).

### ELISA assay

RADA-peptide hydrogel was diluted in 0.1 M sodium bicarbonate buffer (pH 8.6) and adsorbed to black 96-well Maxisorb plates (Nunc, NY) at 4 C overnight. Plates were washed in TBS with 0.1% Tween-20 (TBST), blocked with 10% non-fat dry milk followed by incubation with dilutions of hamster brain homogenate at 4 C for 18 h. Plates were washed, blocked a second time and incubated with m-a-PrP (mAb IPC1; 10K) diluted in TBST with 0.1% IgG-free BSA (Jackson) followed by gt-a-m-HRP (1∶10K) for 1 h. Plates were resolved by ECL (Supersignal Femto, Pierce) and chemiluminescence detected using a Wallac Victor II (PerkinElmer, MA).

### Immunofluorescent microscopy

Hamster brains were fixed in 10% neutral buffered formalin 72 h at 4 C, sunk in 30% sucrose and 5 µm coronal cryosections collected on glass slides. Sections were washed in 10 mM PBS (pH 7.2) with 0.1% Triton-X100, blocked (Superblock, Pierce) and incubated with m-a-PrP (IPC1, 1∶1K) and rbt-a-GFAP (1∶1K; Novus) or ms-a-GFAP (1∶1K; BD Biosciences) and rbt-a-IBA1 (1∶200; Biocare Medical, CA) diluted in PBST-BSA for 2 h. Alexa Fluor-488 gt-a-ms-IgG or Alexa Fluor-568 gt-a-rbt-IgG (Molecular Probes-Invitrogen, CA) diluted 1∶1500 in PBST-BSA containing DAPI and incubated 1 h. Sections were imaged using a Leica SP5 confocal microscope equipped with AOTF/AOBS and blue Argon, Yellow DPSS (561 nm) and 405 nm lasers (Leica, Germany).

### Histochemistry

Hamster brains were fixed and 1 mm coronal sections cut using a steel hamster brain matrix (EM sciences) and gross morphology imaged using a Leica EZ4D stereo microscope. Thin cyrosections were collected stained with CAT Hematoxylin (Biocare Medical), dehydrated in graded-ETOH, cleared in Xylene, and coverslipped using DPX (EM sciences). Sections were imaged using a Leica DMI400B with an attached DFC320 R2 digital camera.

### Statistics

Sigma Stat software was used for statistical analysis (Systat, CA). Kruskal-Wallis one way analysis of variance on ranks was used followed by pairwise comparison using Mann-Whitney U Rank Sum Test.

## References

[1] Prusiner SB (1998). Prions.. Proc Natl Acad Sci U S A.

[2] Bolton DC, McKinley MP, Prusiner SB (1982). Identification of a protein that purifies with the scrapie prion.. Science.

[3] Prusiner SB, Groth D, Serban A, Koehler R, Foster D (1993). Ablation of the prion protein (PrP) gene in mice prevents scrapie and facilitates production of anti-PrP antibodies.. Proc Natl Acad Sci U S A.

[4] DeArmond SJ, Yang SL, Cayetano-Canlas J, Groth D, Prusiner SB (1994). The neuropathological phenotype in transgenic mice expressing different prion protein constructs.. Philos Trans R Soc Lond B Biol Sci.

[5] Mallucci G, Dickinson A, Linehan J, Klohn PC, Brandner S (2003). Depleting neuronal PrP in prion infection prevents disease and reverses spongiosis.. Science.

[6] Gelain F, Horii A, Zhang S (2007). Designer self-assembling peptide scaffolds for 3-d tissue cell cultures and regenerative medicine.. Macromol Biosci.

[7] Nagai Y, Unsworth LD, Koutsopoulos S, Zhang S (2006). Slow release of molecules in self-assembling peptide nanofiber scaffold.. J Control Release.

[8] Ellis-Behnke RG, Liang YX, You SW, Tay DK, Zhang S (2006). Nano neuro knitting: peptide nanofiber scaffold for brain repair and axon regeneration with functional return of vision.. Proc Natl Acad Sci U S A.

[9] Zhao X, Zhang S (2006). Molecular designer self-assembling peptides.. Chem Soc Rev.

[10] Gelain F, Lomander A, Vescovi AL, Zhang S (2007). Systematic studies of a self-assembling peptide nanofiber scaffold with other scaffolds.. J Nanosci Nanotechnol.

[11] Yokoi H, Kinoshita T, Zhang S (2005). Dynamic reassembly of peptide RADA16 nanofiber scaffold.. Proc Natl Acad Sci U S A.

[12] Zhang S, Rich A (1997). Direct conversion of an oligopeptide from a beta-sheet to an alpha-helix: a model for amyloid formation.. Proc Natl Acad Sci U S A.

[13] DeArmond SJ, Prusiner SB (2003). Perspectives on prion biology, prion disease pathogenesis, and pharmacologic approaches to treatment.. Clin Lab Med.

[14] Pan KM, Baldwin M, Nguyen J, Gasset M, Serban A (1993). Conversion of alpha-helices into beta-sheets features in the formation of the scrapie prion proteins.. Proc Natl Acad Sci U S A.

[15] Safar J, Roller PP, Gajdusek DC, Gibbs CJ (1993). Conformational transitions, dissociation, and unfolding of scrapie amyloid (prion) protein.. J Biol Chem.

[16] Scott MR, Supattapone S, Nguyen HO, DeArmond SJ, Prusiner SB (2000). Transgenic models of prion disease.. Arch Virol.

[17] Legname G, Nguyen HO, Peretz D, Cohen FE, DeArmond SJ (2006). Continuum of prion protein structures enciphers a multitude of prion isolate-specified phenotypes.. Proc Natl Acad Sci U S A.

[18] Makarava N, Baskakov IV (2008). The same primary structure of the prion protein yields two distinct self-propagating states.. J Biol Chem.

[19] Hecker R, Taraboulos A, Scott M, Pan KM, Yang SL (1992). Replication of distinct scrapie prion isolates is region specific in brains of transgenic mice and hamsters.. Genes Dev.

[20] DeArmond SJ, Sanchez H, Yehiely F, Qiu Y, Ninchak-Casey A (1997). Selective neuronal targeting in prion disease.. Neuron.

[21] Ludewigs H, Zuber C, Vana K, Nikles D, Zerr I (2007). Therapeutic approaches for prion disorders.. Expert Rev Anti Infect Ther.

[22] Collinge J, Clarke AR (2007). A general model of prion strains and their pathogenicity.. Science.

[23] Soto C, Kascsak RJ, Saborio GP, Aucouturier P, Wisniewski T (2000). Reversion of prion protein conformational changes by synthetic beta-sheet breaker peptides.. Lancet.

[24] Chabry J, Caughey B, Chesebro B (1998). Specific inhibition of in vitro formation of protease-resistant prion protein by synthetic peptides.. J Biol Chem.

[25] De GL, Selvaggini C, Ghibaudi E, Diomede L, Bugiani O (1994). Conformational polymorphism of the amyloidogenic and neurotoxic peptide homologous to residues 106–126 of the prion protein.. J Biol Chem.

[26] Chabry J, Priola SA, Wehrly K, Nishio J, Hope J (1999). Species-independent inhibition of abnormal prion protein (PrP) formation by a peptide containing a conserved PrP sequence.. J Virol.

[27] Kaneko K, Ball HL, Wille H, Zhang H, Groth D (2000). A synthetic peptide initiates Gerstmann-Straussler-Scheinker (GSS) disease in transgenic mice.. J Mol Biol.

[28] Holmes TC, de Lacalle S, Su X, Liu G, Rich A (2000). Extensive neurite outgrowth and active synapse formation on self-assembling peptide scaffolds.. Proc Natl Acad Sci U S A.

[29] Lu X, Lu D, Scully MF, Kakkar VV (2006). Integrins in drug targeting-RGD templates in toxins.. Curr Pharm Des.

[30] Horii A, Wang X, Gelain F, Zhang S (2007). Biological designer self-assembling Peptide nanofiber scaffolds significantly enhance osteoblast proliferation, differentiation and 3-D migration.. PLoS ONE.

[31] Narmoneva DA, Oni O, Sieminski AL, Zhang S, Gertler JP (2005). Self-assembling short oligopeptides and the promotion of angiogenesis.. Biomaterials.

[32] Tashiro K, Sephel GC, Greatorex D, Sasaki M, Shirashi N (1991). The RGD containing site of the mouse laminin A chain is active for cell attachment, spreading, migration and neurite outgrowth.. J Cell Physiol.

[33] Graner E, Mercadante AF, Zanata SM, Forlenza OV, Cabral AL (2000). Cellular prion protein binds laminin and mediates neuritogenesis.. Brain Res Mol Brain Res.

[34] Leucht C, Simoneau S, Rey C, Vana K, Rieger R (2003). The 37 kDa/67 kDa laminin receptor is required for PrP(Sc) propagation in scrapie-infected neuronal cells.. EMBO Rep.

[35] Gauczynski S, Nikles D, El-Gogo S, Papy-Garcia D, Rey C (2006). The 37-kDa/67-kDa laminin receptor acts as a receptor for infectious prions and is inhibited by polysulfated glycanes.. J Infect Dis.

[36] Caughey B, Race RE (1992). Potent inhibition of scrapie-associated PrP accumulation by congo red.. J Neurochem.

[37] Demaimay R, Harper J, Gordon H, Weaver D, Chesebro B (1998). Structural aspects of Congo red as an inhibitor of protease-resistant prion protein formation.. J Neurochem.

[38] Caspi S, Halimi M, Yanai A, Sasson SB, Taraboulos A (1998). The anti-prion activity of Congo red. Putative mechanism.. J Biol Chem.

[39] Roterman I, Krol M, Nowak M, Konieczny L, Rybarska J (2001). Why Congo red binding is specific for amyloid proteins - model studies and a computer analysis approach.. Med Sci Monit.

[40] Lee KS, Linden R, Prado MA, Brentani RR, Martins VR (2003). Towards cellular receptors for prions.. Rev Med Virol.

[41] Solassol J, Crozet C, Lehmann S (2003). Prion propagation in cultured cells.. Br Med Bull.

